# Survey data on climate change adaptation and barriers to adoption among smallholder farmers in Nepal

**DOI:** 10.1016/j.dib.2021.107620

**Published:** 2021-11-20

**Authors:** Prahlad Lamichhane, Kelly K. Miller, Michalis Hadjikakou, Brett A. Bryan

**Affiliations:** Centre for Integrative Ecology (CIE), School of Life and Environmental Sciences, Deakin University, Burwood, Victoria, Australia

**Keywords:** Resilience, Smallholder agriculture, Climate change, Adaptation, Nepal

## Abstract

The dataset presents the raw data collected through household surveys of smallholder farmers on adaptation to climatic variabilities and change in Sudurpaschim Pradesh (Far Western Province), Nepal. The dataset comprises farmers' responses on the likely determinants of adaptation decisions, actual uptake of adaptation measures, and the barriers to adaptation. We collected the data by conducting face-to-face interviews of 327 farmers using structured questionnaires in all nine districts representing the Mountain, Hill, and Terai agroecosystems in the province. We employed a stratified random sampling technique to recruit participants and interviewed them during December 2019 and March 2020. The interview methodology was approved by the Human Ethics Review Committee at Deakin University, Australia. The dataset is important for understanding the drivers of climate change adaptation and the barriers to adaptation to enhance the resilience of smallholder agriculture in far-western Nepal and can inform climate change adaptation strategies for the region and for the smallholder agroecosystems more broadly. The data are provided with this article.

## Specifications Table

**Table:** 

Subject	Environmental Sciences
Specific subject area	Resilience and climate change adaptation in smallholder agriculture
Type of data	Categorical and numerical data presented in tables and figures Excel file
How data were acquired	Data were acquired through face-to-face interviews of smallholder farmers using a structured questionnaire in the Nepali language. The questionnaire is provided as a supplementary file.
Data format	Raw, Analysed Dataset in Microsoft Excel (.xlsx) file format.
Parameters for data collection	The data were obtained from nine districts of the Sudurpaschim Pradesh, Nepal. 327 smallholder farmers were recruited from each district for a face-to-face interview that satisfied the following eligibility criteria: (a) willingness to participate, (b) have at least five years of farming experience, and (c) be aged >18 years.
Description of data collection	Data were collected from a household survey using structured questionnaires in Sudurpaschim Pradesh, Nepal. Questionnaire was developed and administered in Nepali language. Given lower level of literacy among farmers in the study area, face-to-face interviews were conducted by researchers and a trained surveyor with extensive socio-ecological understanding of the study area. Given socio-ecological heterogeneity of the study area, stratified random sampling technique was used to recruit the respondents. The administrative divisions (i.e. district, municipality, and ward) form the strata. Two municipalities from each district were randomly selected, and then a ward from the selected municipalities was randomly selected for farmer's recruitment for interviews to better represent the study area. Using Election Commission's voters' list, potential interviewees were randomly selected for the wards and were recruited for the face-to-face interview using structured questionnaire that satisfy the recruitment criteria.
Data source location	Sudurpaschim Pradesh, Nepal. The province extends between 28°30″—30°03″ N Latitude and 80°03″—81°25″ E Longitude (Fig. 1) that encompasses the Mountain, Hill and Terai agroecosystems.
Data accessibility	With the article

## Value of the Data

• The dataset is important to understand smallholder farmers' adaptation to climatic variabilities and adaptation barriers in the socio-ecologically heterogeneous agroecosystem of far-western Nepal.
• The dataset benefits stakeholders such as policymakers and practitioners in government and non-governmental organisations as it offers a detailed account of farmer's perception, barriers, and adaptation decisions that could be used in developing programs and inform strategies for enhancing adaptation and resilience in smallholder agriculture both at local and regional levels.
• The dataset may be used for a comparative assessment of adaptation, including the knowledge and perception of farmers, across heterogeneous smallholder agroecosystems. In addition, the dataset may be used to evaluate the adaptation policy effectiveness in the future as the dataset may form a baseline for longitudinal adaptation research in smallholder agriculture.
• Beyond climate change adaptation, with smallholder agriculture being the economic mainstay of the study area, the dataset could be relevant for various other purposes, including local/regional planning, community development and livelihood promotion programmes and research at local and regional levels.

## 1 Data Description

**Fig. 1:**
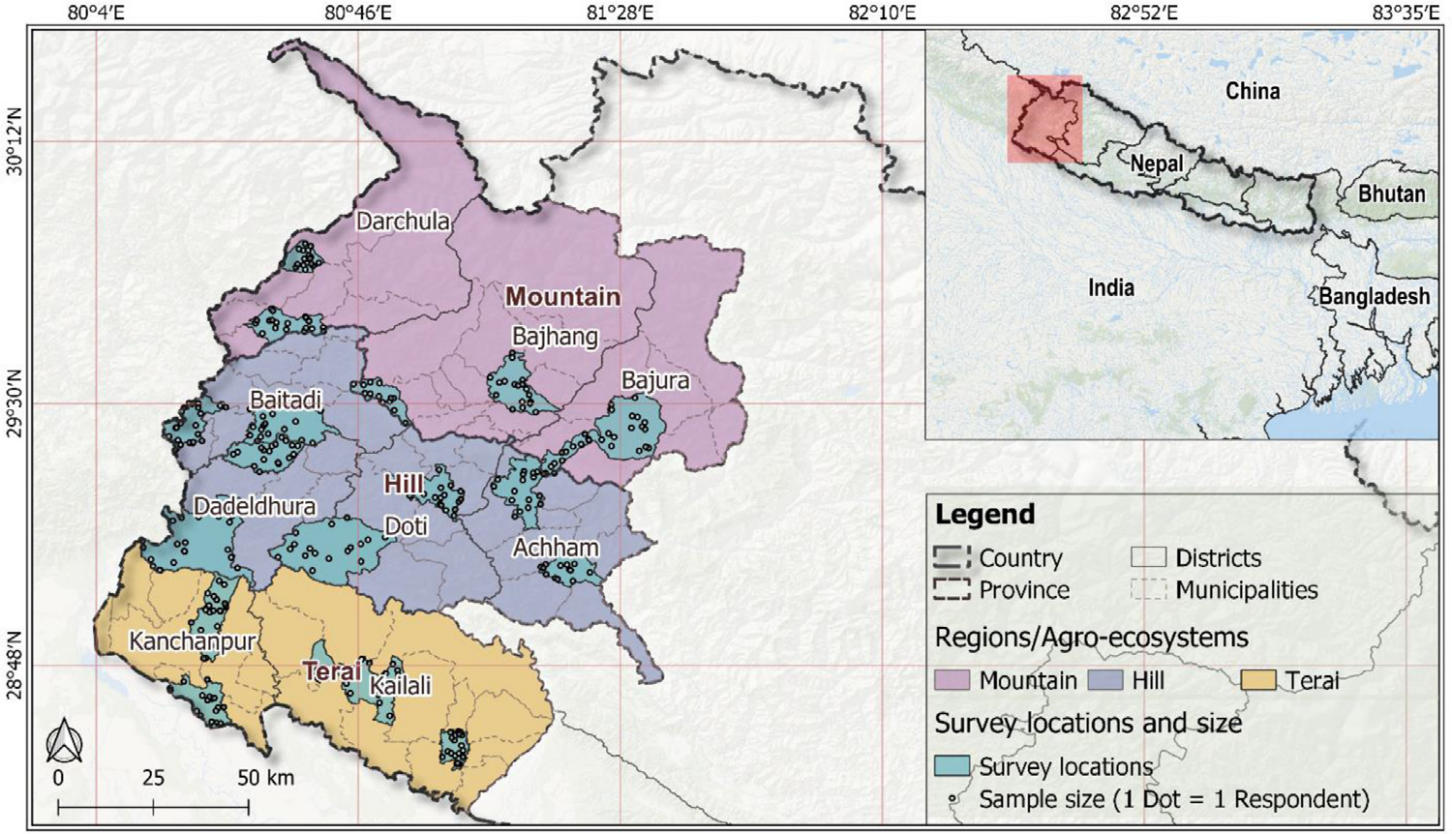
**Study area, sampling locations, and the sample size.** Respondents were recruited from the Mountain, Hill, and Terai agroecosystems.

The dataset contains the responses of 327 smallholder farmers in Far Western Province, Nepal, collected through face-to-face interviews (Table 1, Fig. 1). Smallholder agriculture is the province's mainstay and engages a significant proportion of the population in the sector [1,2]. Climate change is evident and has already impacted smallholder agriculture in the province [3]. Most of the districts in the province are chronically food insecure [4], and climatic change has further exacerbated food insecurity [5]. This dataset captures farmers' resilience, adaptation, and barriers to climate change adaptation in smallholder agriculture in the province and could inform adaptation policy.

**Table 1:** Interviewees recruited for the survey in the study area. Interviewees were recruited from the Mountain, Hill and Terai agroecosystems in the study area.

Study area	Agroecosystem	Number of interviewees	Proportion (%)
Far Western Province, Nepal (Fig. 1)	Mountain	100	30.58
	Hill	148	45.26
	Terai	79	24.16

The dataset contains: (a) Socio-economic and demographic characteristics, (b) Smallholder farm characteristics, (c) Smallholder farm management practices, (d) Farmers perceptions on climatic impacts/risk and its management, and (e) adoption of climate change adaptation measures and barriers to adoption.

### 1.1 Socio-economic and demographic characteristics

**Fig. 2:**
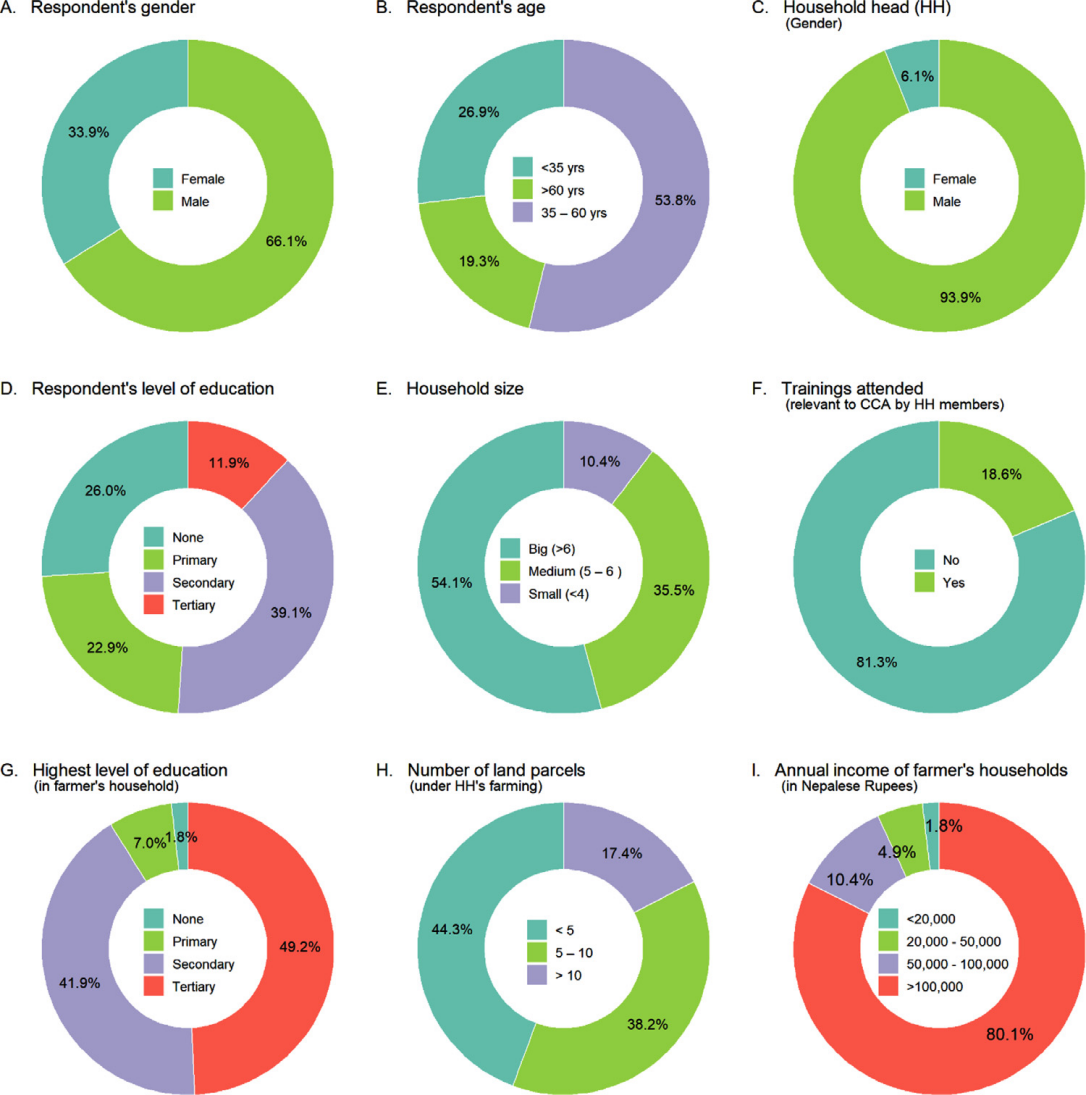
Socio-demographic characteristics of the respondents in the survey.

The socio-economic characteristics of the respondents and the household were collected using multiple-choice questions, and they include characteristics including gender (sex), age, education, training, and the household income (Fig. 2). Data are provided as a supplementary file.

### 1.2 Smallholder farm characteristics

Attributes, including the farm size, crops grown, affiliations, income, and agricultural markets characterising the study participants was collected using multiple-choice responses and is presented in Table 2. Data are provided as a supplementary file.

**Table 2:** Smallholder characteristics *(n = 327).*

Characteristics	Category	Frequency	Proportion (%)
Farm size	< 0.5 ha	194	59.33
	0.5 – 1.0 ha	83	25.38
	1.0 – 1.5 ha	35	10.70
	1.5 – 2.0 ha	12	3.67
	> 2ha	3	0.92

Households growing crops	Rice	314	96.02
	Wheat	323	98.78
	Maize	229	70.03

Farming experience	Less than 10 years	21	6.42
	More than 10 years	306	93.58

Land ownership (tenure)	Outright ownership	165	50.46
	Partly	126	38.53
	None	36	11.00

Food sufficiency from smallholder	Suffice	175	52.52
	Insufficient	152	46.58

Sell cereal crop production	Sell production	86	26.30
	Self-consumption	241	73.70

Production of cash crop	Produce cash crops	100	30.58
	Do not produce cash crop	227	69.42

Cash generation from livestock	Yes	89	13.46
	No	238	86.54

Off-farm income	Yes	245	74.92
	No	82	25.08

Investment of off-farm income in agriculture	Yes	114	46.53
	No	131	53.47

Market distance	< 5 km	64	19.57
	5 – 10 km	147	44.95
	> 10 km	116	35.47

Cooperative group membership	Yes	74	22.63
	No	253	77.37

Farmers' group membership	Yes	111	33.45
	No	216	66.05

Labour intensiveness	Labour intensive	251	76.76
	Labour + machinery	76	23.24

### 1.3 Smallholder farm management

This section presents the farmers' responses associated with irrigation, fertilisation, and selection of the crop varieties in smallholder agriculture. Tables 3 and 4 present data on irrigation frequency in rice, wheat and maize, and irrigation coverage in smallholdings. Irrigation systems in the study area are dependent upon monsoon rainfall [6]. Table 5 reports the dependency of irrigation systems on monsoon rainfall. Table 6 presents the use of soil moisture conservation practices in the study area.

**Table 3:** Frequency of irrigation in Rice, Wheat, and Wheat.

Crop	Crop growth stages	2 times a week	Once a week	Once in 10 days	Once in >10 days	No irrigation
Rice	Early-stage	169	72	10	0	63
	Mid-stage	157	80	10	4	63
	Late-stage	64	75	94	8	73

Wheat	Early-stage	0	0	16	136	171
	Mid-stage	0	0	11	114	198
	Late-stage	0	0	6	147	170

Maize	No reported irrigation for Maize at any stage of crop development.

**Table 4:** Irrigation coverage during crop seasons (% of landholding).

Crop	No irrigable land	Under 25%	25 - under 50%	50 – Under 75%	75% and above
Rice	63	34	27	84	106
Wheat	144	21	56	43	59
Maize	No reported irrigation for Maize.

**Table 5:** Irrigation system's dependency on monsoon rain.

Level of dependency	Responses	Proportion (%)
Dependent	257	78.59
Partly independent	40	12.23
Independent	30	9.17

**Table 6:** Soil moisture management practice.

Practice	Responses	Proportion (%)
Mulching	21	6.42
Framing in terrace with shoulder bund	201	61.47
Rainwater harvest for use in the dry season	34	10.40
Hedgerow/Agroforestry	109	33.33
Others (e.g., reduced tillage, tillage scheduling, planting stabilisation grass along the terrace bund, and sprinkler irrigation)	32	9.79

More than 93% (*n* = 305) of the respondents stated that they change crop varieties. However, such change often occurs between locally available crop varieties rather than via the introduction of new varieties. Table 7 presents farmers’ responses relating to the change in crop varieties and the reasons for change. 64% of the farmers reported mixed cropping practice in smallholdings.

**Table 7:** Rationale of change in crop variety.

Reasons	Responses	Proportion (%)
New variety is high yielding variety	291	88.99
New variety performs good in less rainfall	9	2.75
New variety can tolerate more droughts	70	21.41
New variety has a short crop cycle	115	35.17
New variety has a better market value	10	3.06
New variety has a better nutritional value	30	9.17
Other reasons (farmer reported other reasons include the availability of seed, past success/failure experiences for change in variety, cultural practices, and the community decisions)	70	21.41

The data on farmers' perceived land fertility and fertiliser application is presented in Tables 8 and 9, respectively. Table 10 presents the farmers' perceived suitability of their smallholding for growing major cereal crops. Table 11 illustrates smallholder farmers' dependency on external resources and inputs.

**Table 8:** Perceived land fertility for cropping.

Perceived fertility	Responses	Proportion (%)
Good	84	25.68
Average	229	70.04
Poor	14	4.28

**Table 9:** Households applying manure and chemical fertilisers in rice, wheat, and maize.

Crops	Manure application		Fertiliser application	
	Yes	No	Yes	No
Rice (n = 314)	291	23	104	210
Wheat (n = 323)	312	11	165	155
Maize (n = 229)	216	13	64	165

**Table 10:** Perceived suitability of land for various crops.

	Crops		
Perceived fertility	Rice	Wheat	Maize
Suitable	129	88	46
Moderately suitable	168	228	217
Not suitable	30	11	10
No response	0	0	54

**Table 11:** Smallholder's dependence on external resources/inputs.

Degree of dependence	Number of responses	Proportion (%)
Hardly any	67	20.49
Some degree	216	66.05
Everything	44	13.46

### 1.4 Farmers’ perceptions on climatic impacts/risk and its management

**Fig. 3:**
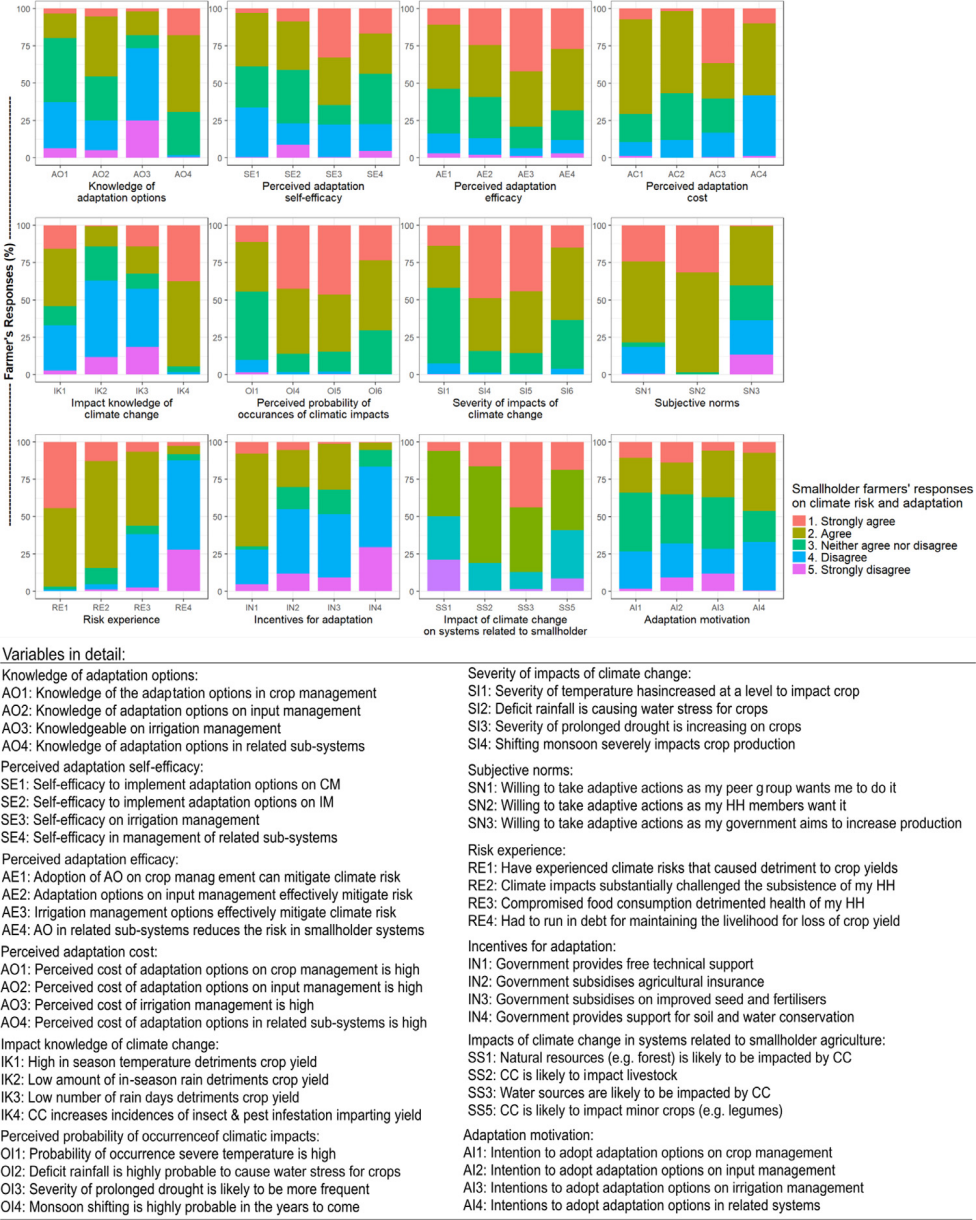
Farmers' response on climatic impacts/risk and management.

The details of farmers’ adaptation knowledge, self-efficacy, adaptation effectiveness, adaptation cost, impact knowledge, probability of occurrence, the severity of occurrence, subjective norms, risk experience, incentives, impacts on related systems, and adaptation motivation were measured using indicators and are illustrated in Fig. 3. Data are provided as a supplementary file.

### 1.5 Adoption of adaptation measures and barriers to adoption

**Fig. 4:**
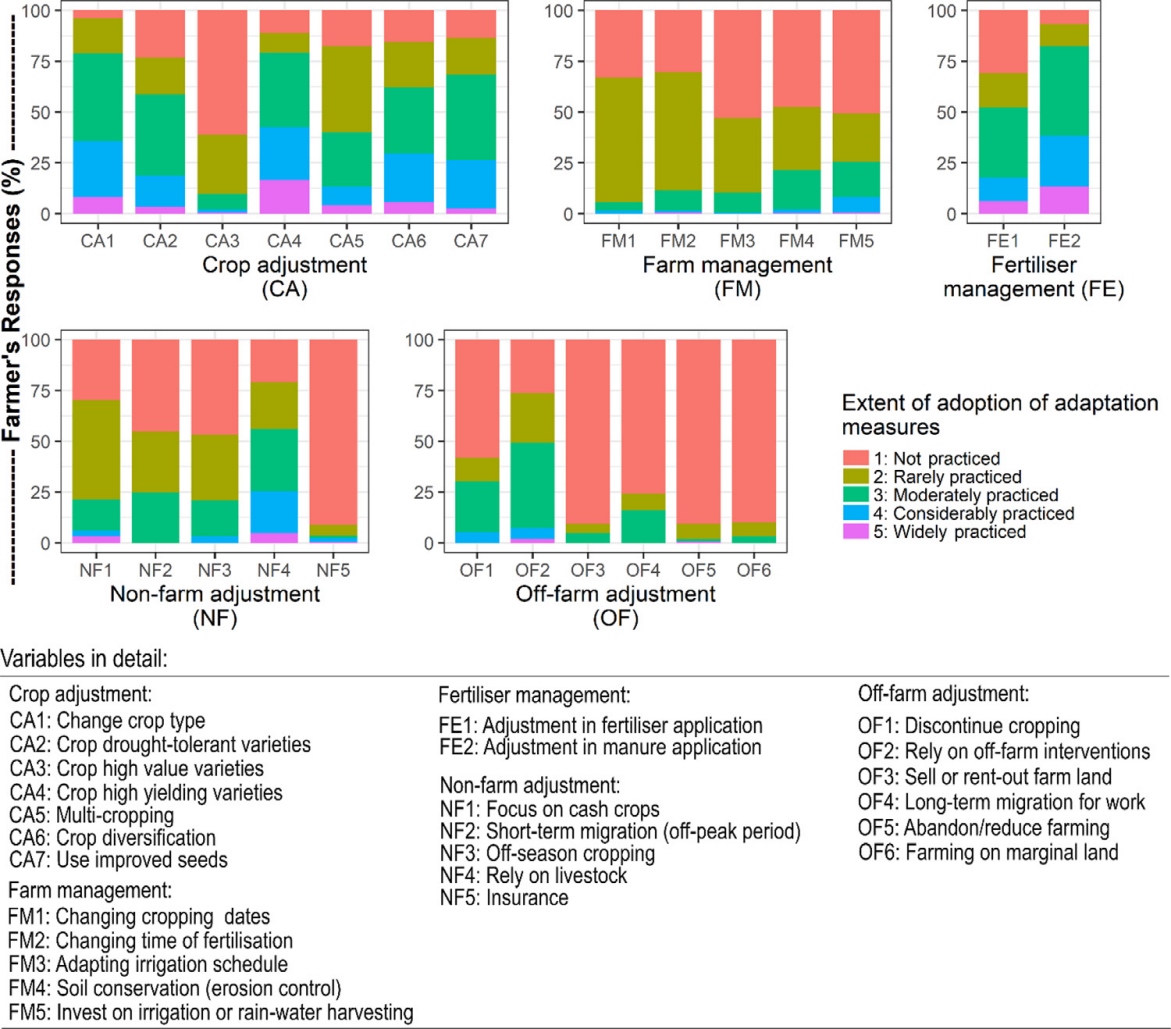
Farmers' responses on the degree of adoption of adaptation measures.

**Fig. 5:**
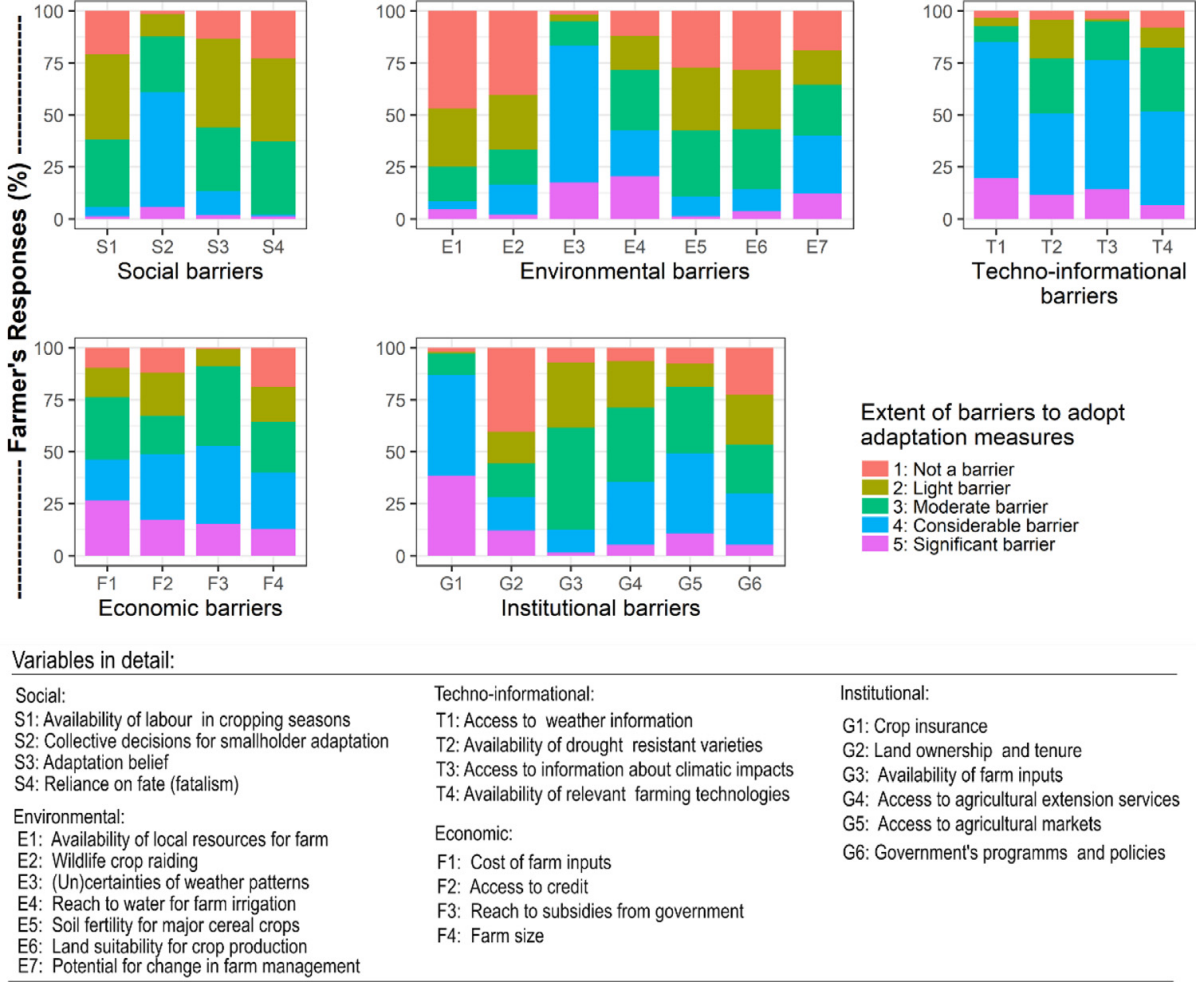
Farmers' response on the extent of barriers associated with adaptation measures.

The research collected smallholder farmers’ adoption of the adaptation measures for crop adjustment, farm management, fertiliser management, non-farm adjustments, and off-farm adjustments in 1-5 Likert scale (Fig. 4). In addition, farmers’ responses on barriers to adaptation associated with social, techno-informational, economic, environmental and institutional barriers measured in 1-5 Likert scale are illustrated in (Fig. 5).

## 2 Experimental Design, Materials and Methods

**Fig. 6:**
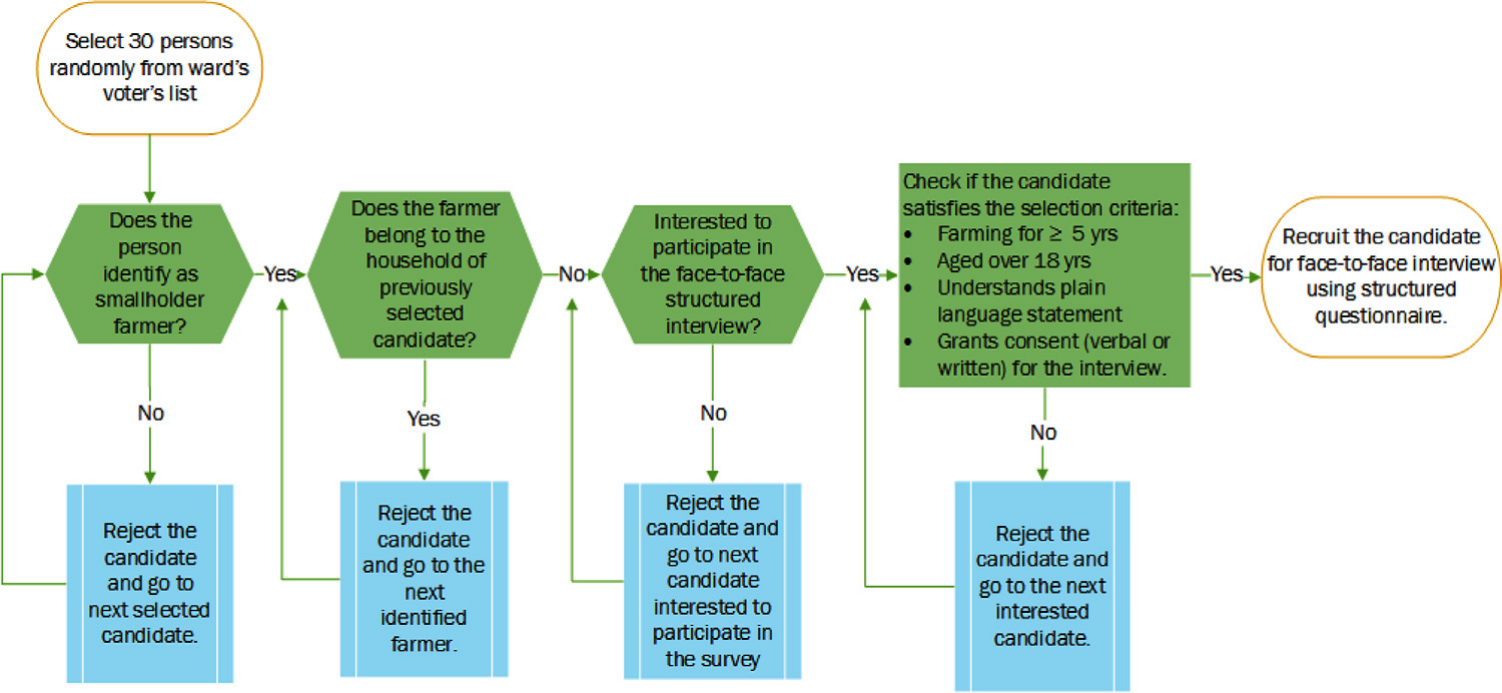
Participant selection process for face-to-face interview; only 15–20 participants were recruited from each ward (N = 18) in the study area for interviews.

The dataset was constructed based on face-to-face interviews with a total of 327 smallholder farmers from the three agroecosystems in the Far Western Province, Nepal. We used a stratified random sampling approach to recruit respondents for the interview [6]. We first identified the survey locations and then selected participants from those identified survey locations for the face-to-face interview using a structured questionnaire. Given the environmental and socio-economic heterogeneity of the study area, survey locations were allocated across all districts (N = 9) in the study area (Fig. 1) to recruit participants representing the heterogeneous agroecosystems [7]. We randomly selected two municipalities from each district (N = 18), and then a ward from the selected municipalities was randomly selected for farmers' interviews (N = 18). Our approach of stratified multi-stage random recruitment of respondents better represents the heterogeneous study area [6], [7], [8]. We acquired a list of residents from ward offices based on the Election Commission's voters' list. Then, we randomly selected potential interviewees from the selected wards that satisfy the recruitment criteria (Fig. 6). Along with a willingness to participate, farmers with at least five years of farming experience and at least 18 years of age were eligible to participate in the survey. We contacted the potential respondents, enquired about their interest to participate in the survey (supplied a copy of the Plain Language Statement), and confirmed their participation by collecting their consent to participate. Consent to participate in the interview was obtained verbally or in writing based on the preference of the participant. The respondents were the household heads and/or their representatives. A questionnaire containing both closed and open-ended questions was used to interview the respondent. Open-ended questions were designed to collect the narratives behind the responses [9,10]. The 5-point Likert scale was used to quantify subjective responses, e.g., the perceptions, knowledge, or the behavioural interests associated with climate change adaptation, adoption of adaptation measures, and the extent of barriers to adaptation. Questions designed to draw socio-demographic information and farm management practices were mainly alternative-choice type questions. Data were entered into Microsoft Excel and imported to R for visualisation [11].

## Ethics Statement

Ethical approval was obtained from the Faculty of Science, Engineering and Built Environment Human Ethics Advisory Group, Deakin University (ref. no. STEC-43-2018-LAMICHHANE). Consent to participate in the interview was acquired, verbally or in writing based on participant's choice, from all participants. Respondent identities are completely anonymised in the dataset. Qualitative responses collected during the field survey are not incorporated in the dataset to ensure the anonymity of the respondents to the fullest extent.

## CRediT Author Statement

**Prahlad Lamichhane:** Conceptualization, Methodology, Data curation, Visualization, Writing – original draft; **Kelly K. Miller:** Conceptualization, Methodology, Writing – review & editing; **Michalis Hadjikakou:** Conceptualization, Methodology, Writing – review & editing; **Brett A. Bryan:** Conceptualization, Methodology, Writing – review & editing.

## Declaration of Competing Interest

The authors declare that they have no known competing financial interests or personal relationships which have or could be perceived to have influenced the work reported in this article.

## Appendix Supplementary materials

Image, application 1

Image, application 2
