# Supplementary material for: Survey data on climate change adaptation and barriers to adoption among smallholder farmers in Nepal
Source: Data Brief. 2021 Nov 20;39:107620. doi: 10.1016/j.dib.2021.107620 (PMC8633867; doi:10.1016/j.dib.2021.107620)
Supplement: Supplementary file 1 [file mmc1.docx]

QUESTIONNAIRE

*To be filled by the surveyor:*

District [ ___________________ ] Municipality [ ________________ ] Ward [ ____ ]

*To be filled by the participant or surveyor based on the participants response:*

1. **FARM CHARACTERISTICS**
2. What are the major crops you grow? Tick all that apply.

Rice □, Maize □, Wheat □, Buckwheat □, Other(______________) □

1. What area of land do you crop? Tick the relevant box.

| For Terai | For Hills and Mountain |
| --- | --- |
| Under 0.5 ha □, 0.5 - 1 ha □, 1 - 1.5 ha □, 1.5-2 ha □, above 2 ha □ | Under 10 ropani □, <10 – 20 ropani □, >20 - 30 ropani □, >30 – 40 ropani □, above 40 ropani □ |

1. For how many years have you been farming? Please tick the relevant box.

Under 5 years □, 5 years – 10 years □, More than 10 years

1. On what fraction of your farm do you grow the following crops? Tick the relevant box.

|  | Under 25 % | 25% – under 50% | 50% – under 75% | 75% and above |
| --- | --- | --- | --- | --- |
| Rice | □ | □ | □ | □ |
| Wheat | □ | □ | □ | □ |
| Maize | □ | □ | □ | □ |

1. Do you own your farm (land ownership) outright? All □, Partly □, None □
2. Do you keep livestock? Yes □, No □. If No, go to Q8.
3. How many head of each type of livestock do you run? Please mention the number in the spaces provided.

Buffalo [ ], Cow [ ], Goat/Sheep [ ], Chicken [ ], Pig [ ], Other__________ [ ]

1. Does your farm’s cereal crop production suffice your household’s food and livestock feed needs? Yes □, No □
2. Do you sell cereal products from your farm? Yes □, No □ in …………………….. .
3. Do you grow cash crops that directly go to market other than the cereals? Yes □, No □
4. Do you receive cash from livestock? Yes □, No □
5. Do you have any off-farm incomes? Yes □, No □
6. Do you invest off-farm income in agricultural practices? Yes □, No □
7. Can you access credit sources for farming practices? Yes □, Yes, to some extent □, No □. If yes, where from?.............................. .
8. How far is the input purchase market (e.g. seeds, fertilizers) from your farm/house? [______] km
9. If you sell any products to market, how far is the output market? □ km
10. Are you a member of any local community cooperatives? Yes □, No □
11. Do you belong to any farmer’s groups in the community? Yes □, No □
12. What kind of farming do you practice? labour-intensive □, mechanised □
13. **FARM MANAGEMENT PRACTICES**
14. What are the cropping schedules for the following crops? Please provide the date/time-window.

| Crop | Sowing/ transplanting | Harvesting |
| --- | --- | --- |
| Rice |  |  |
| Wheat |  |  |
| Maize |  |  |

1. Do you irrigate crops? Yes □, No □, if no go to question Q25.
2. What is typical irrigation schedule for your farm (interval of subsequent irrigation in days)?

| Crop | Growth Phase (early season) | Maturity Phase (mid-season) | Senescence Phase (late season) |
| --- | --- | --- | --- |
| Rice |  |  |  |
| Wheat |  |  |  |
| Maize |  |  |  |

1. What fraction of land has reliable irrigation facility for different crops?

| Crop | No Irrigation | Under 25 % | 25% – under 50% | 50% – under 75% | 75% and above |
| --- | --- | --- | --- | --- | --- |
| Rice | □ | □ | □ | □ | □ |
| Wheat | □ | □ | □ | □ | □ |
| Maize | □ | □ | □ | □ | □ |

1. Is the source of irrigation water independent of monsoon rain? Yes □, Moderately independent □, No □
2. Do you practice any measures to preserve soil moisture in your farm? Tick all that apply.

Mulching □, Dikes □, Pond for rainwater harvesting □, Agroforestry □, Other: [____________]

1. Do you apply fertilizers or chemicals on your farm? Yes □, No □, if no go to question Q28­.
2. Please provide detailed estimates of the manure, and chemical fertilizers you normally apply for different crops?

| Crop | Manure application | | Chemical fertiliser application | |
| --- | --- | --- | --- | --- |
|  | Time schedule | Amount | Time schedule | Amount |
| Rice |  |  |  |  |
| Wheat |  |  |  |  |
| Maize |  |  |  |  |

Amount: in kg/area unit, if local unit conversion to kg and ha, type: type of input

1. What crop varieties do you cultivate? Are they different from the varieties that you have cultivated in the past? (Note down the name of varieties even if the respondent gives the local names.)

| Crop | Currently cultivation crop varieties | Varieties cultivated in the past |
| --- | --- | --- |
| Rice |  |  |
| Wheat |  |  |
| Maize |  |  |

1. If you have changed the crop varieties, why did you do so? Circle the responses that apply.
2. Change adopted because the new variety is high yielding variety
3. Change adopted because the new variety performs good in less rainfall
4. Changes adopted because the new variety can tolerate more droughts
5. Changes adopted because the new variety has short crop cycle
6. Changes adopted because the new variety has a better market value
7. Changes adopted because the new variety has a better nutritional value
8. If other, please mention:___________________________________________________________
9. How do you rate the soil fertility of your farm? Good □, Average □, Poor □
10. What do you think about the suitability of your farm to grow the following crops?

| Crop | Suitable | Moderately suitable | Not Suitable |
| --- | --- | --- | --- |
| Rice |  |  |  |
| Wheat |  |  |  |
| Maize |  |  |  |

1. Do you do inter-cropping or mixed cropping? Yes □, No □
2. What is the aim of mixed-cropping or inter-cropping? Circle all that apply.
3. Maximising the land productivity
4. Enrich nutrients to the main crop (e.g. inter/mix cropping nitrogen fixing crops)
5. Provide shade to the main crop
6. Control the loss of top soil
7. If other, please mention:______________________________
8. **PERCEPTION OF CLIMATE CHANGE**
9. Do you believe the statement that 'climate change is happening?' Please circle the response below.

1: I don’t believe the statement

2: I have lower level of belief on the statement

3: The statement might be true

4: I believe on the statement

5: I strongly belief on the statement

1. **Considering the past 20 years, how do you compare current temperature to the temperature in the past?** Please circle the response that best represents your experience.

*(1: Temperature getting substantially cooler, 3: not changed, 5: temperature getting substantially warmer)*

| Statements | | | | | |
| --- | --- | --- | --- | --- | --- |
| Maximum temperature | 1 | 2 | 3 | 4 | 5 |
| Minimum temperature | 1 | 2 | 3 | 4 | 5 |

1. **Considering the past 20 years, how do you compare current rainfall to the rainfall in the past?** Please circle the response that best represents your experience. *(1: Rainfall substantially decreased, 3: not changed, 5: Rainfall substantially increased)*

| Statements | | | | | |
| --- | --- | --- | --- | --- | --- |
| Rainfall amount | 1 | 2 | 3 | 4 | 5 |
| Days with rain | 1 | 2 | 3 | 4 | 5 |

1. **Considering the past 20 years, how do you feel about changes in droughts?** Please circle the response that best represent your experience. *(1: Substantially increased, 3: not changed, 5: substantially decreased)*

| Statements | | | | | |
| --- | --- | --- | --- | --- | --- |
| Number of drought events | 1 | 2 | 3 | 4 | 5 |
| Duration of drought spells | 1 | 2 | 3 | 4 | 5 |

1. **Considering the past 20 years, how did you feel about the extremities of changes?** Please circle the response that best represent your experience. *(1: Substantially increased, 3: not changed, 5: substantially decreased)*

| **Statements** | | | | | |
| --- | --- | --- | --- | --- | --- |
| Extreme hot days | 1 | 2 | 3 | 4 | 5 |
| Extreme cold days | 1 | 2 | 3 | 4 | 5 |
| Days with extremely heavy rains | 1 | 2 | 3 | 4 | 5 |
| Severe droughts | 1 | 2 | 3 | 4 | 5 |
| Flood events leading to inundation | 1 | 2 | 3 | 4 | 5 |

1. **Considering the past 20 years, how do you feel about climate variability? Please circle the response that best represents your experience.** *(1 = Strongly disagree, 2 = Disagree, 3 = Undecided, 4 = Agree, 5 = Strongly agree)*

| **Statements** | | | | | |
| --- | --- | --- | --- | --- | --- |
| I feel that the onset of rainy season in getting less predictable. | 1 | 2 | 3 | 4 | 5 |
| I feel that the retreat of rainy season in getting less predictable. | 1 | 2 | 3 | 4 | 5 |
| I feel that the temporal distribution of rainfall is getting less predictable. | 1 | 2 | 3 | 4 | 5 |

1. **KNOWLEDGE OF CLIMATE CHANGE IMPACTS ON CROPS**
2. **Over last 20 years, how do you perceive the impact of climate change on crops and yields?** Please circle the responses which most accurately reflects the extent to which you agree or disagree with each statement.

| Statements | Strongly disagree | Disagree | Undecided | Agree | Strongly agree |
| --- | --- | --- | --- | --- | --- |
| High in-season temperature increases yield. | 1 | 2 | 3 | 4 | 5 |
| Low amount of in-season rain detriments crop yield | 1 | 2 | 3 | 4 | 5 |
| Low number of rain days detriments crop yield | 1 | 2 | 3 | 4 | 5 |
| Climate change increases incidences of insect and pest infestation imparting yield | 1 | 2 | 3 | 4 | 5 |

1. **CLIMATE RISK EXPERIENCE**

| 1. **How do you feel about your experience of weather induced or climatic risks?** Please circle the responses which most accurately reflects the extent to which you agree or disagree with each statement. | Strongly disagree | Disagree | Undecided | Agree | Strongly agree |
| --- | --- | --- | --- | --- | --- |
| I have experienced climate risks that caused detriment to crop yields | 1 | 2 | 3 | 4 | 5 |
| Climate impacts substantially challenged the subsistence of my family | 1 | 2 | 3 | 4 | 5 |
| Compromised food consumption detriment health condition of my family | 1 | 2 | 3 | 4 | 5 |
| I had to run in debt for maintaining the livelihood for loss of crop yield | 1 | 2 | 3 | 4 | 5 |

1. **SUBJECTIVE NORMS AND GOVERNMENT POLICIES**

| 1. **How do you feel about the expectations of other people and government related to you regarding your climate change adaptation actions?**   Please circle the responses which most accurately reflects the extent to which you agree or disagree with each statement. | Strongly disagree | Disagree | Undecided | Agree | Strongly agree |
| --- | --- | --- | --- | --- | --- |
| Willing to take adaptive actions as my peer group wants me to do it | 1 | 2 | 3 | 4 | 5 |
| Willing to take adaptive actions as my family members want me to do it | 1 | 2 | 3 | 4 | 5 |
| Willing to take adaptive actions as my government aims to increase production | 1 | 2 | 3 | 4 | 5 |
| I should conduct adaptive measures since government policies requires me do that. | 1 | 2 | 3 | 4 | 5 |

1. **INCENTIVES**

| 1. **How do you feel about the available incentives for climate change adaptation actions to smallholder farmers?** Please circle the responses which most accurately reflects the extent to which you agree or disagree with each statement. | Strongly disagree | Disagree | Undecided | Agree | Strongly agree |
| --- | --- | --- | --- | --- | --- |
| The government provides free technical support in identifying resilient crop varieties for my land | 1 | 2 | 3 | 4 | 5 |
| Government subsidises agricultural insurance to protect farmers from climate impacts | 1 | 2 | 3 | 4 | 5 |
| Government subsidises on improved seed and fertilisers to the smallholder farmers to adapt to climate change impacts. | 1 | 2 | 3 | 4 | 5 |
| Government provides financial and material supports for soil and water conservation to deal with the water stress and drought | 1 | 2 | 3 | 4 | 5 |

1. **PERCEIVED PROBABILITY OF OCCURRENCE OF RISK EVENTS**
2. **How do you perceive the probability of occurrence of events that are likely to impact your smallholder farming practices in the future?** Please circle the responses which most accurately reflects the extent to which you perceive probabilities of each events.

| **Climate risk events** | **Perceived probability of occurrences** |
| --- | --- |
| Probability of occurrence severe temperature is high | 1 2 3 4 5 |
| Deficit rainfall is highly probable to cause water stress for crops | 1 2 3 4 5 |
| Severity of prolonged drought is likely to be more frequent | 1 2 3 4 5 |
| Monsoon shifting is highly probable in the years to come | 1 2 3 4 5 |

Probability of occurrences: 1 = Strongly disagree, 2 = Disagree, 3 = Undecided, 4 = Agree, 5 = Strongly agree

1. **PERCEIVED SEVERITY OF CLIMATIC RISK IMPACTS ON CROPS**
2. **How do you perceive the severity of events that are likely to impact your smallholder practice in future?** Please circle the responses which most accurately reflects the extent to which you perceive probabilities of each events.

| **Climate risk events** | **Perceived severity of impact on crops** |
| --- | --- |
| Severity of temperature has increased at a level to impact crop | 1 2 3 4 5 |
| Deficit rainfall is causing water stress for crops | 1 2 3 4 5 |
| Severity of prolonged drought is increasing on crops | 1 2 3 4 5 |
| Shifting monsoon severely impacts crop production | 1 2 3 4 5 |

Severity of events: 1 = Strongly disagree, 2 = Disagree, 3 = Undecided, 4 = Agree, 5 = Strongly agree

1. **IMPACTS ON SYSTEMS RELATED TO SMALLHOLDER AGRICULTURE**
2. **How do you perceive the likely impact of future climate to the following related systems?** Please circle the responses which most accurately reflects your perception.

| **Sub-systems related to smallholder systems** | **Strongly disagree** | **Disagree** | **Undecided** | **Agree** | **Strongly agree** |
| --- | --- | --- | --- | --- | --- |
| Natural resources (e.g. forest) is likely to be impacted by climate change | 1 | 2 | 3 | 4 | 5 |
| Climate change is likely to impact livestock | 1 | 2 | 3 | 4 | 5 |
| Water sources are likely to be impacted by climate change | 1 | 2 | 3 | 4 | 5 |
| Climate change is likely to impact minor crops (e.g. legumes) | 1 | 2 | 3 | 4 | 5 |

1. **KNOWLEDGE, EFFICACY, COST, AND INTENTIONS TO ADAPTION**
2. **How do you feel about the knowledge, efficacy, cost and intentions to the following adaptation actions? Please circle the respective numbers.**

| **Have knowledge of adaptation options** | Strongly Agree ←⎯⎯→ Strongly Disagree | | | | |
| --- | --- | --- | --- | --- | --- |
| Knowledge of the adaptation options in crop management | 1 | 2 | 2 | 4 | 5 |
| Knowledge of adaptation options on input management | 1 | 2 | 2 | 4 | 5 |
| Knowledgeable on irrigation management | 1 | 2 | 2 | 4 | 5 |
| Knowledge of adaptation options in related sub-systems | 1 | 2 | 2 | 4 | 5 |
| **Perceived Self-efficacy to practice Adaptation** | Extremely High ←⎯⎯⎯→ Extremely Low | | | | |
| Self-efficacy to implement adaptation options on crop management | 1 | 2 | 2 | 4 | 5 |
| Self-efficacy to implement adaptation options on irrigation management | 1 | 2 | 2 | 4 | 5 |
| Self-efficacy on irrigation management | 1 | 2 | 2 | 4 | 5 |
| Self-efficacy in management of related sub-systems | 1 | 2 | 2 | 4 | 5 |
| **Perceived Adaptation Efficacy** | Strongly Agree ←⎯⎯→ Strongly Disagree | | | | |
| Implementation of adaptation options on crop management can mitigate climate risk | 1 | 2 | 2 | 4 | 5 |
| Adaptation options on input management are effective to mitigate risk | 1 | 2 | 2 | 4 | 5 |
| Irrigation management options are effective in mitigating climate risk | 1 | 2 | 2 | 4 | 5 |
| Adaptation options on related sub-systems reduces the risk in smallholder systems | 1 | 2 | 2 | 4 | 5 |
| **Perceived Adaptation Cost** | Strongly Agree ←⎯⎯→ Strongly Disagree | | | | |
| Perceived cost of adaptation options on crop management is high | 1 | 2 | 2 | 4 | 5 |
| Perceived cost of adaptation options on input management is high | 1 | 2 | 2 | 4 | 5 |
| Perceived cost of irrigation management is high | 1 | 2 | 2 | 4 | 5 |
| Perceived cost of adaptation options in related sub-systems is high | 1 | 2 | 2 | 4 | 5 |

1. **ADAPTATION BARRIERS**
2. **How do you feel about the following dimensions as a barrier to the adaptation?** Please circle the most relevant response.

| Barriers | Not a barrier | Light barrier | Moderate barrier | Considerable barrier | Significant barrier |
| --- | --- | --- | --- | --- | --- |
| **Social barriers** | | | | | |
| Availability of farm labour during peak agricultural operations | 1 | 2 | 3 | 4 | 5 |
| Collective decisions among smallholders to adaptation | 1 | 2 | 3 | 4 | 5 |
| Adaptation belief | 1 | 2 | 3 | 4 | 5 |
| Reliance on fate (fatalism) | 1 | 2 | 3 | 4 | 5 |
| **Economic Barriers** | | | | | |
| Cost of farm inputs | 1 | 2 | 3 | 4 | 5 |
| Access to credit | 1 | 2 | 3 | 4 | 5 |
| Reach to govt subsidy | 1 | 2 | 3 | 4 | 5 |
| Farm size for big investments | 1 | 2 | 3 | 4 | 5 |
| **Environmental Barriers** | | | | | |
| Availability of local resources for farm | 1 | 2 | 3 | 4 | 5 |
| Wildlife crop raiding | 1 | 2 | 3 | 4 | 5 |
| (Un)certainity of weather patterns | 1 | 2 | 3 | 4 | 5 |
| Access to water for irrigation | 1 | 2 | 3 | 4 | 5 |
| Soil fertility for major cereal crops | 1 | 2 | 3 | 4 | 5 |
| Land suitability for cereal production | 1 | 2 | 3 | 4 | 5 |
| Potential for change in farm management | 1 | 2 | 3 | 4 | 5 |
| **Governance and Institutional Barriers** | | | | | |
| Crop insurance | 1 | 2 | 3 | 4 | 5 |
| Land ownership/ tenure | 1 | 2 | 3 | 4 | 5 |
| Availability of farm inputs | 1 | 2 | 3 | 4 | 5 |
| Availability of ag. extension services | 1 | 2 | 3 | 4 | 5 |
| Access to agricultural markets | 1 | 2 | 3 | 4 | 5 |
| Government programs and policies | 1 | 2 | 3 | 4 | 5 |
| **Techno Informational Barriers** | | | | | |
| Access to timely weather information | 1 | 2 | 3 | 4 | 5 |
| Knowledge about drought resistant crop varieties | 1 | 2 | 3 | 4 | 5 |
| Access to information about climatic impacts | 1 | 2 | 3 | 4 | 5 |
| Availability of appropriate farming technologies |  |  |  |  |  |

1. **ADAPTATION DECISIONS**
2. What are the key actions/decisions you have practiced to adapt to climatic impacts? Please circle the responses that most accurately represent your actions.

| Adaptation actions | Not practiced | Rarely practiced | Practiced in part | Well practiced | Practiced in full scale |
| --- | --- | --- | --- | --- | --- |
| **Crop Adjustment** |  |  |  |  |  |
| Change in crop type (varieties) | 1 | 2 | 3 | 4 | 5 |
| Crop drought-tolerant varieties | 1 | 2 | 3 | 4 | 5 |
| Crop high value varieties | 1 | 2 | 3 | 4 | 5 |
| Crop high yielding varieties | 1 | 2 | 3 | 4 | 5 |
| Multi-cropping | 1 | 2 | 3 | 4 | 5 |
| Crop diversification | 1 | 2 | 3 | 4 | 5 |
| Improved seed | 1 | 2 | 3 | 4 | 5 |
| **Farm Management** |  |  |  |  |  |
| Changing planting dates | 1 | 2 | 3 | 4 | 5 |
| Changing timing of fertilisation | 1 | 2 | 3 | 4 | 5 |
| Adapting irrigation schedule | 1 | 2 | 3 | 4 | 5 |
| Soil conservation & erosion control | 1 | 2 | 3 | 4 | 5 |
| Invest on Irrigation/rainwater harvesting | 1 | 2 | 3 | 4 | 5 |
| **Fertilisation Management.** |  |  |  |  |  |
| Adjustment in fertiliser application | 1 | 2 | 3 | 4 | 5 |
| Adjustment in manure application | 1 | 2 | 3 | 4 | 5 |
| **Non-farm Adjustments** |  |  |  |  |  |
| Cash crops | 1 | 2 | 3 | 4 | 5 |
| Seasonal migration (during crop offseason) | 1 | 2 | 3 | 4 | 5 |
| Off-season cropping | 1 | 2 | 3 | 4 | 5 |
| Rely more on livestock | 1 | 2 | 3 | 4 | 5 |
| Insurance | 1 | 2 | 3 | 4 | 5 |
| **Off-farm Adjustments** |  |  |  |  |  |
| Discontinue cropping | 1 | 2 | 3 | 4 | 5 |
| Rely more on off-farm interventions | 1 | 2 | 3 | 4 | 5 |
| Selling/rent-out farm land | 1 | 2 | 3 | 4 | 5 |
| Long-term migration (aboard/city/India) | 1 | 2 | 3 | 4 | 5 |
| Abandon cultivating less profitable crops | 1 | 2 | 3 | 4 | 5 |
| Farming on marginal land | 1 | 2 | 3 | 4 | 5 |

1. **SOCIO-DEMOGRAPHIC INFORMATION:**

Please tick the relevant boxes.

1. Which age group do you belong to?

Age: 18 – 35 years □, Above 35 years – under 60 years □, 60 years and above □

1. What is your gender? Male □, Female □, Other □
2. Who heads your household? Male □, Female □, Other □
3. What is your household size? Four or less □, 5-6 □, 7 and above □
4. What is your level of education? No formal education □, Primary education □, Secondary school □, Tertiary □
5. Have you ever attended in the trainings related to climate change adaptation in the past three years? Yes □, No □, if yes, how many? □
6. What is the highest level of education that your family member attained?

No formal education □, Primary education □, Secondary school □, Tertiary □

1. How many members of your family above 18 work in other occupations? [ ], if any answer Q67.
2. Where are these members working? district □, major cities in NP □, India □, abroad □
3. How many parcels of land do you farm on? Please tick the relevant box.

Les than 5 □, 5 – 10 □, 10 and more □

1. What is the level of your household’s annual earnings? (in Nepalese Rupees)

No monetary income □, Under 20,000 □, 20,000 - 50,000 □, 50,000-100,000 □, >100,000 □

----------------------- THE END -----------------------
